# PCK1-mediated glycogenolysis facilitates ROS clearance and chemotherapy resistance in cervical cancer stem cells

**DOI:** 10.1038/s41598-024-64255-6

**Published:** 2024-06-13

**Authors:** Xinxin Chen, Nan Yang, Ying Wang, Shuang Yang, Yuanhong Peng

**Affiliations:** 1 Department of Cadre Ward 2, the Second Affiliated Hospital of Harbin Medical University, Harbin, 150086 China; 2 Department of Cadre Ward 1, the Second Affiliated Hospital of Harbin Medical University, Harbin, 150086 China

**Keywords:** PCK1, Reactive oxygen species, Chemotherapy resistance, Cervical cancer, Cancer stem cells, Cancer therapy, Cancer therapeutic resistance, Biochemistry, Cancer, Cell biology

## Abstract

Cervical cancer, one of the most common gynecological cancers, is primarily caused by human papillomavirus (HPV) infection. The development of resistance to chemotherapy is a significant hurdle in treatment. In this study, we investigated the mechanisms underlying chemoresistance in cervical cancer by focusing on the roles of glycogen metabolism and the pentose phosphate pathway (PPP). We employed the cervical cancer cell lines HCC94 and CaSki by manipulating the expression of key enzymes PCK1, PYGL, and GYS1, which are involved in glycogen metabolism, through siRNA transfection. Our analysis included measuring glycogen levels, intermediates of PPP, NADPH/NADP^+^ ratio, and the ability of cells to clear reactive oxygen species (ROS) using biochemical assays and liquid chromatography–mass spectrometry (LC–MS). Furthermore, we assessed chemoresistance by evaluating cell viability and tumor growth in NSG mice. Our findings revealed that in drug-resistant tumor stem cells, the enzyme PCK1 enhances the phosphorylation of PYGL, leading to increased glycogen breakdown. This process shifts glucose metabolism towards PPP, generating NADPH. This, in turn, facilitates ROS clearance, promotes cell survival, and contributes to the development of chemoresistance. These insights suggest that targeting aberrant glycogen metabolism or PPP could be a promising strategy for overcoming chemoresistance in cervical cancer. Understanding these molecular mechanisms opens new avenues for the development of more effective treatments for this challenging malignancy.

## Introduction

Cervical cancer is one of the most common gynecological malignancies worldwide. According to the World Health Organization, over half a million women are diagnosed with cervical cancer each year, and approximately 250,000 women die from this disease^1^. Cervical cancer is closely associated with infection by human papillomavirus (HPV), particularly high-risk types, such as HPV16 and HPV18^2,3^. These viruses are primarily transmitted through sexual contact, including skin-to-skin and mucosal contact during sexual activity. The risk factors for cervical cancer include early sexual debut, multiple sexual partners, immunodeficiency, and smoking^4,5^.

Clinical chemotherapy is a common treatment modality for cervical cancer. Commonly used chemotherapeutic agents include platinum-based drugs (e.g., cisplatin and carboplatin), taxanes (e.g., paclitaxel and docetaxel), 5-fluorouracil (5-FU), and docetaxel^6^. Chemotherapy resistance is a significant challenge in cervical cancer treatment, as cancer cells gradually lose sensitivity to chemotherapeutic drugs. Understanding the molecular mechanisms underlying cervical cancer resistance, including aberrant regulation of signaling pathways and changes in cellular responses, is crucial for developing new treatment strategies and improving the effectiveness of cervical cancer treatment.

Glutathione and thioredoxin are two major cellular antioxidant systems that function to clear intracellular ROS (reactive oxygen species), relying on nicotinamide adenine dinucleotide phosphate (NADPH) generated through the pentose phosphate pathway (PPP) as an electron donor. Zhou et al. suggested that in tumor cells, G6PD primarily contributes to antioxidant defense, rather than other enzymes such as cytosolic malic enzymes (ME1, -2, and -3) or methylenetetrahydrofolate dehydrogenase 2 (MTHFD2). This provides a more accurate representation of the current understanding of NADPH generation in tumor cells^7^. Previous studies have revealed that carbon and sulfur derived from glycogen breakdown can enter the PPP and generate NADPH, providing reducing power for ROS clearance to maintain the survival of immune cells^8^. Recent research has revealed the accumulation of glycogen in drug-resistant tumor cell lines and patient tumor tissues^9,10^. Inhibition of glycogen breakdown metabolism or the PPP pathway results in a significant increase in ROS levels in drug-resistant tumor cells^11^. These findings suggest that drug-resistant tumor stem cells rely on the glycogen-PPP pathway to supply NADPH and respond to chemotherapy. Therefore, we hypothesize that in drug-resistant tumor stem cells, glycogen breakdown drives PPP to produce NADPH in response to chemotherapy-induced ROS, thereby maintaining cell survival and promoting chemoresistance. Modulation of abnormal glycogen metabolism may provide a potential strategy to reverse tumor cell resistance.

## Method and materials

### Study approval

Cervical cancer sample collection was approved by the Clinical Research Ethics Committee of Harbin Medical University and conducted in accordance with relevant ethical regulations. Informed consent was obtained from each patient, and the study protocol (no. 202327) was approved by the Clinical Research Ethics Committee of Harbin Medical University and complied with all relevant ethical guidelines. Animal procedures were performed in accordance with protocols approved by the Institutional Animal Care and Use Committee (IACUC) of Harbin Medical University (1-2020122002-0012). All experiments were performed according to the relevant guidelines, regulations, and ARRIVE guidelines.

### Cell and reagent

The HCC94 and CaSki human cervical cancer cell lines were obtained from the American type culture collection (Manassas, VA, USA). The cells were cultured in DMEM (Dulbecco’s modified Eagle’s medium) supplemented with 10% fetal bovine serum, 2 mM l-glutamine, 100 U/mL penicillin, and 100 µg/mL streptomycin. The cell cultures were maintained in a standard incubator at 37 °C with 5% CO_2_. DMEM without glucose and fetal bovine serum was purchased from Gibco (Grand Island, NY, USA). C13-labeled glucose was obtained from Sigma–Aldrich.

### Oxidative stress assay

HCC94 and CaSki cells were seeded at a density of 5 × 105 cells/mL and treated with cisplatin (a negative control solution) for 48 h. To assess cellular oxidative stress, cells were stained with 5 µL of 2ʹ,7ʹ-dichlorofluorescein diacetate (Sigma–Aldrich, USA) diluted in DMSO. The fluorescence intensity was measured using a fluorescence microplate reader.

### siRNA transfection

siRNAs targeting human PCK1, GYS1, and PYGL mRNA and an RNAi-negative control siRNA (siNC) were obtained from Obio Scientific, Inc. (Shanghai, China). Transfection was conducted using Lipofectamine RNAiMax (Thermo, catalog number: 13778030) and opti-MEM medium (Thermo, catalog number: 31985062) following the manufacturer’s instructions using 5 nM of siRNA. The presented data represent the results of three independent experiments, each performed in triplicates. siRNA transfection in vivo was achieved by encapsulating cholesterol-modified siRNA into lipid nanoparticles (LNPs). These LNPs were then injected directly into the tumor tissue via a local injection. This approach allows for efficient delivery of siRNA to tumor cells, enhancing the knockdown effect of the targeted genes.

### NADPH/ NADP^+^ quantification

The NADP^+^/NADPH ratio was determined in cell lysates using the NADPH NADP^+^ quantification colorimetric kit (Abcam, catalog number: ab65349), according to the manufacturer’s instructions. Colorimetric measurements were performed at 450 nm using a Hidex Sense 96-well plate reader.

### Tumor models

NSG mice (6–8 weeks old) were purchased from Charles River Laboratories (China) and maintained in a pathogen-free facility supplied with sterile food and water. Tumor models were established by subcutaneously injecting HCC94 and CaSki human cancer cells (5 × 10^6^ cells per mouse) into 6–8 week-old NSG mice. The total number total number of mice used was 30. All animal experiments were conducted in three independent replicates. To minimize individual variations, 6–8 age- and sex-matched mice were included in each group (n = 5). Cisplatin (2 mg/kg) was intravenously injected into mice with HCC94 or CaSki tumors 14 days after the tumor injection. Tumor size was monitored over time and tumor-induced lethality was defined as a tumor that reached 225 mm^2^ in area. All efforts were made to minimize animal suffering and to reduce the number of animals used. Mice were anesthetized with isoflurane (1–5%) for induction and maintenance of anesthesia for 24 h after the last stimulation. The mice were subsequently sacrificed using CO_2_ and the material was collected.

### Cell viability assay

Cells were seeded at a density of 5 × 10^4^ cells/well in triplicate. In some experiments, cells were seeded at a density of 2.5 × 10^4^ cells per well in triplicate. The following day, the cells were incubated with 20 mg/mL 3-(4,5-dimethylthiazol-2-yl)-2,5-diphenyltetrazolium bromide (MTT) for 3 h. The absorbance was then measured at 570 nm.

### RNA extraction and quantitative real-time PCR

Total RNA extraction was performed using TRIzol reagent (Invitrogen, Cat. No. 15596026), according to the manufacturer’s protocol. RNA was reverse transcribed into complementary DNA (cDNA) using SYBR Green master mix reagent (Applied Biosystems, Cat. No. 4309155). RT-qPCR was performed using SYBR Green real-time PCR reagent in the CFX96^™^ real-time system (Bio-Rad). The PCR conditions were as follows: 94 °C for 15 s, 60 °C for 10 s, and 72 °C for 20 s, for 40 cycles. Primers were designed using the NCBI online tool Primer-BLAST (www.ncbi.nlm.nih.gov/tools/primer-blast) as follows: PYGL, forward: 5ʹ- CAGCCTATGGATACGGCATTC-3ʹ, reverse: 5ʹ-CGGTGTTGGTGTGTTCTACTTT-3ʹ; GYS1, forward: 5ʹ- GCGCTCACGTCTTCACTACTG-3ʹ, reverse: 5ʹ- TCCAGATGCCCATAAAAATGGC-3ʹ; PCK1, forward: 5ʹ- AAAACGGCCTGAACCTCTCG-3ʹ, reverse: 5ʹ- ACACAGCTCAGCGTTATTCTC-3ʹ; glyceraldehyde-3-phosphate dehydrogenase (GAPDH), forward: 5ʹ-CATCAAGAAGGTGGTGAAGCAG-3ʹ, reverse: 5ʹ-CGTCAAAGGTGGAGGAGTGG-3ʹ. GAPDH was used as an internal control for mRNA, and the relative gene expression was calculated using the 2^−ΔΔCt^ method.

### Co-immunoprecipitation (Co-IP) assay

For Co-IP assays, samples were lysed and transferred to a 1.5EP tube, followed by overnight incubation at 4 °C with IgG or IP antibodies. Next, the suspended Protein A/G beads were incubated with sample-antibody complexes at 4 °C for 2 h. The IP supernatant was discarded. The beads were then centrifuged and washed five times with TBST. SDS-PAGE sample loading buffer was added to the immunoprecipitants and the mixture was boiled for 10 min at 100 °C. Immunoprecipitated and input proteins were detected using western blot analysis.

### Western blot analysis

Total protein from the tissues or cultured cells was extracted using RIPA lysis buffer containing the protease inhibitor PMSF (Thermo, USA). Approximately equal amounts of protein were separated by 10% SDS-PAGE and transferred to a polyvinylidene fluoride (PVDF) membrane. After blocking with 5% skim milk for 5 min, the membrane was incubated with primary antibodies overnight at 4 °C, with β-actin as an internal control. The next day, membranes were incubated with HRP-conjugated secondary antibodies at room temperature for 1 h. Finally, chemiluminescence detection was performed using an ECL kit to visualize the western blot bands. The primary antibodies included β-actin (13E5, CST), PYGL (ab223788, Abcam), p-PYGL (EPR20881-72, Abcam), PCK1 (16754-1-AP, Protein Tech), and PP1c (ab245664, Abcam). The secondary antibodies included HRP-conjugated goat anti-mouse IgG (SA00001-1, Protein Tech) and HRP-conjugated goat anti-rabbit IgG (SA00001-2, Protein Tech). To conserve both antibodies and nitrocellulose membrane materials, the blots were cut prior to hybridization with antibodies. Original images of blots are provided in Figs. S3–8.

### Patient cohort

We collected 12 cervical cancer specimens from patients who received chemotherapy as a definitive treatment for biopsy-proven cervical cancer at our institution between November 2020 and January 2023. A summary of the patient characteristics is presented in Table 1.

### Immunofluorescence analysis

Immunofluorescence (IF) was performed on formalin-fixed paraffin-embedded (FFPE) cervical cancer tumor tissues to assess protein expression. Anti-human phosphor-PYGL (Abcam) was used as primary antibody. Negative controls were prepared using blocking buffer with non-immune rabbit or mouse serum instead of the primary antibody. After developing in TSA Plus Working Solution for 5 min, the slides were counterstained with DAPI and mounted for confocal analysis.

### LC–MS analysis

For the detection and analysis of R5P, S7P, and 6-PGA, cells were treated with chemotherapeutic drugs for 24 h.

The cells were washed twice in saline and lysed in an extraction solvent (80% methanol/water) for 30 min at − 80 °C. After centrifugation at 12000×*g* for 10 min at 4 °C, the supernatant extracts were analyzed using LC–MS. All the metabolite assay samples were calibrated for protein quantification.

The LC–MS portion of the platform was based on HPLC (Vanquish Horizon UHPLC System, Thermo Fisher Scientific) and a Q Exactive Mass Spectrometer (Thermo Fisher Scientific). LC used the following two analytical methods. (i) The samples were separated on an Xbridge amide column (130 Å, 2.1 mm inner diameter, 100 mm length; waters). Mobile phase A consisted of 20 mM ammonium acetate and 15 mM ammonium hydroxide in water with 3% acetonitrile (pH 9.0), and mobile phase B consisted of acetonitrile. The linear gradient was as follows: 0 min, 85% B; 1.5 min, 85% B, 5.5 min, 30% B; 8 min, 30% B; 10 min, 85% B; 12 min, 85% B. The flow rate was 0.2 mL/min. (ii) Metabolites were separated on a 150 mm × 2.1 mm, 2.7 mm Acquity UPLC BEH C18 column (waters) with a gradient of solvent A (5 mM n,n-dimethyloctylamine, H_2_O, pH 5.5) and solvent B (5 mM n,n-dimethyloctylamine, 90% methanol/H_2_O, pH 5.5). The gradient was 0 min, 10% B; 1.5 min, 10% B; 5.5 min, 100% B; 8 min, 100% B; 10 min, 10% B; 15 min, 10% B; 0.3 mL/min. Sample volumes of 5 mL were injected for LC–MS analysis. Data analysis was performed using the Xcalibur Qual browser software. Peak areas were integrated and corrections for natural abundance were applied. We normalized the metabolite results by the protein amount.

### Quantification and statistical analysis

Statistical analyses were performed using GraphPad Prism 6 and Excel software. Student’s *t*-test was used for comparisons between two groups, whereas one-way ANOVA followed by the post hoc Tukey test was used for multiple group comparisons. Differences in tumor growth were analyzed using two-way ANOVA. Statistical significance was defined as p < 0.05. Data are presented as the mean ± SEM, and significance levels are denoted as *P < 0.05, **P < 0.01, and ***P < 0.001 in the figures.

## Result

### Timely ROS clearance contributes chemotherapy resistance in cancer stem cells

**Figure 1:**
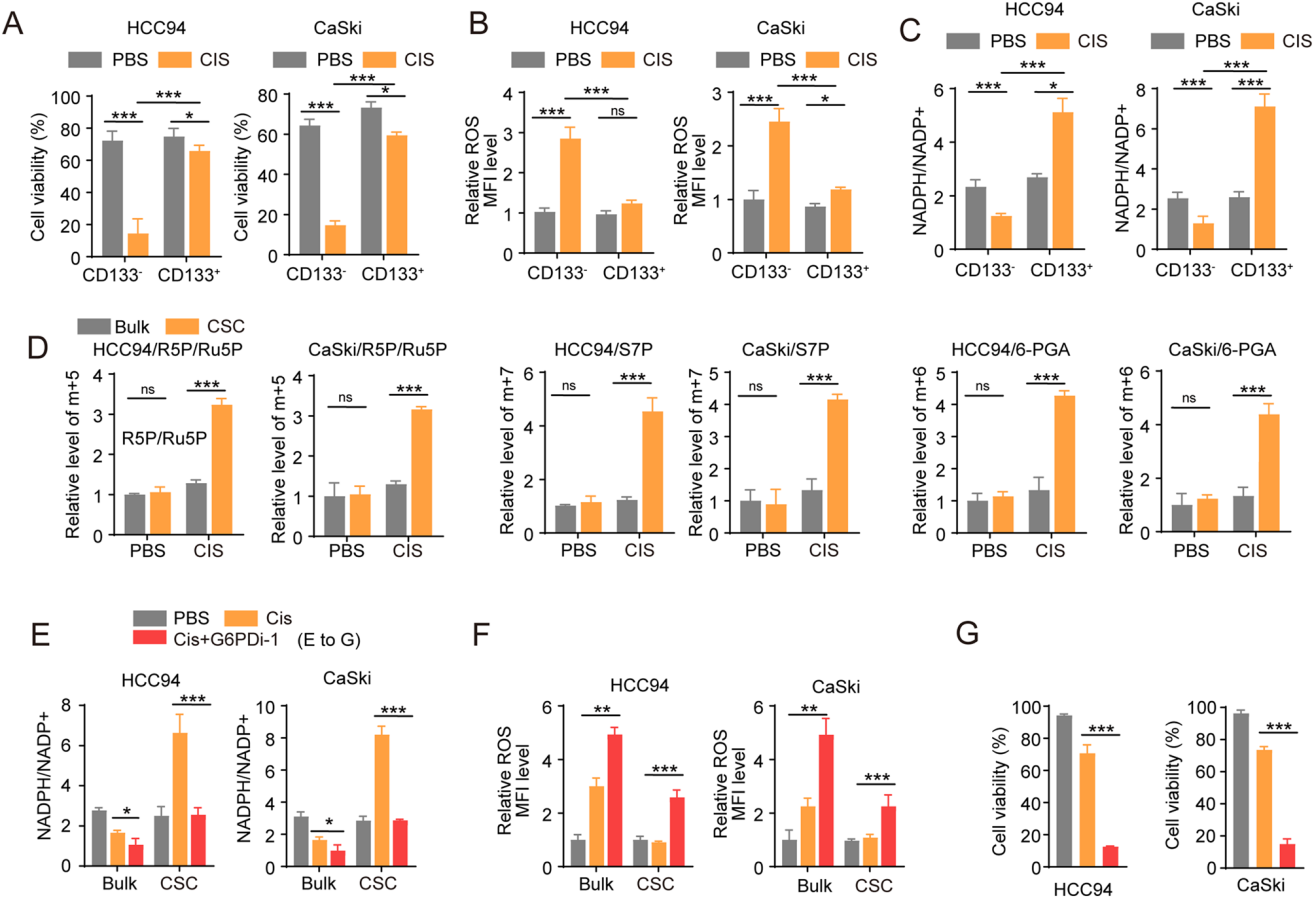
(**A**–**C**) HCC94, and CaSki human cervical cancer cells were sorted into two fractions: CD133^+^ and CD133^−^. After treatment with cisplatin (50 μM) for 48 h, measurements were taken for cell viability (**A**), NADPH/NADP^+^ ratio (**B**), and reactive oxygen species (ROS) levels (**C**). (**D**) HCC94 and CaSki human cancer stem cells or bulk cells were treated with PBS and cisplatin (50uM) for 48 h. The levels of R5P/Ru5P, S7P and 6-PGA (6-phosphogluconic acid) were measured. (**E**–**G**) HCC94, and CaSki human cancer stem cells or bulk cells were treated with cisplatin (50 μM) or G6PDi (50 μM) for 48 h. NADPH (**E**), ROS (**F**), and cell viability (**G**) levels were measured. Data are presented as mean ± SEMs of three independent experiments. ***P < 0.001. Two-tailed student’s *t*-test (**A**–**D**) or one-way analysis of variance (ANOVA) followed by Bonferroni’s test (**E**–**G**).

According to reports, the timely clearance of reactive oxygen species (ROS) generated by chemotherapy drugs plays a crucial role in determining the efficacy of chemotherapy^12^. Cancer stem cells (CSCs) often mediate chemotherapy resistance in various types of tumors^13^. Therefore, we investigated whether CSCs mediate chemotherapy resistance in cervical cancer by regulating ROS clearance. In this study, we used CD133 sorting to isolate CSCs derived from HCC94 or CaSki cells and found consistent resistance to cisplatin compared to CD133^−^ cells (Fig. 1A). Our further observations indicated that the upregulated ROS levels in CSCs under chemotherapy treatment were lower than those in bulk cells (Fig. 1B), suggesting that CSCs may have evolved an intrinsic mechanism to effectively eliminate ROS during chemotherapy, and the rapid clearance of ROS may be a mechanism for tumor stem cells to survive during drug treatment. Interestingly, the NADPH/NADP^+^ ratio in CSCs increased following drug treatment (Fig. 1C). The pentose phosphate pathway (PPP) is an important source of NADPH production^14^. Interestingly, we also observed an increase in the labeled metabolites of the PPP pathway, 6-phosphogluconic acid (6-PGA), R5P/ Ru5P and S7P, in CSCs compared to bulk cells under drug treatment (Fig. 1D and Fig. S1A). The use of the PPP pathway inhibitor G6PDi disrupted the increased NADPH/NADP^+^ ratio in CSCs (Fig. 1E), accompanied by a sharp increase in ROS levels (Fig. 1F) and the disappearance of chemoresistance in cells (Fig. 1F, G). In conclusion, these results suggest that CSCs utilize an activated PPP pathway to rapidly generate NADPH in response to chemotherapeutic drugs, leading to the development of chemoresistance.

### PYGL phosphorylation activates glycogenolysis-PPP in CSCs

**Figure 2:**
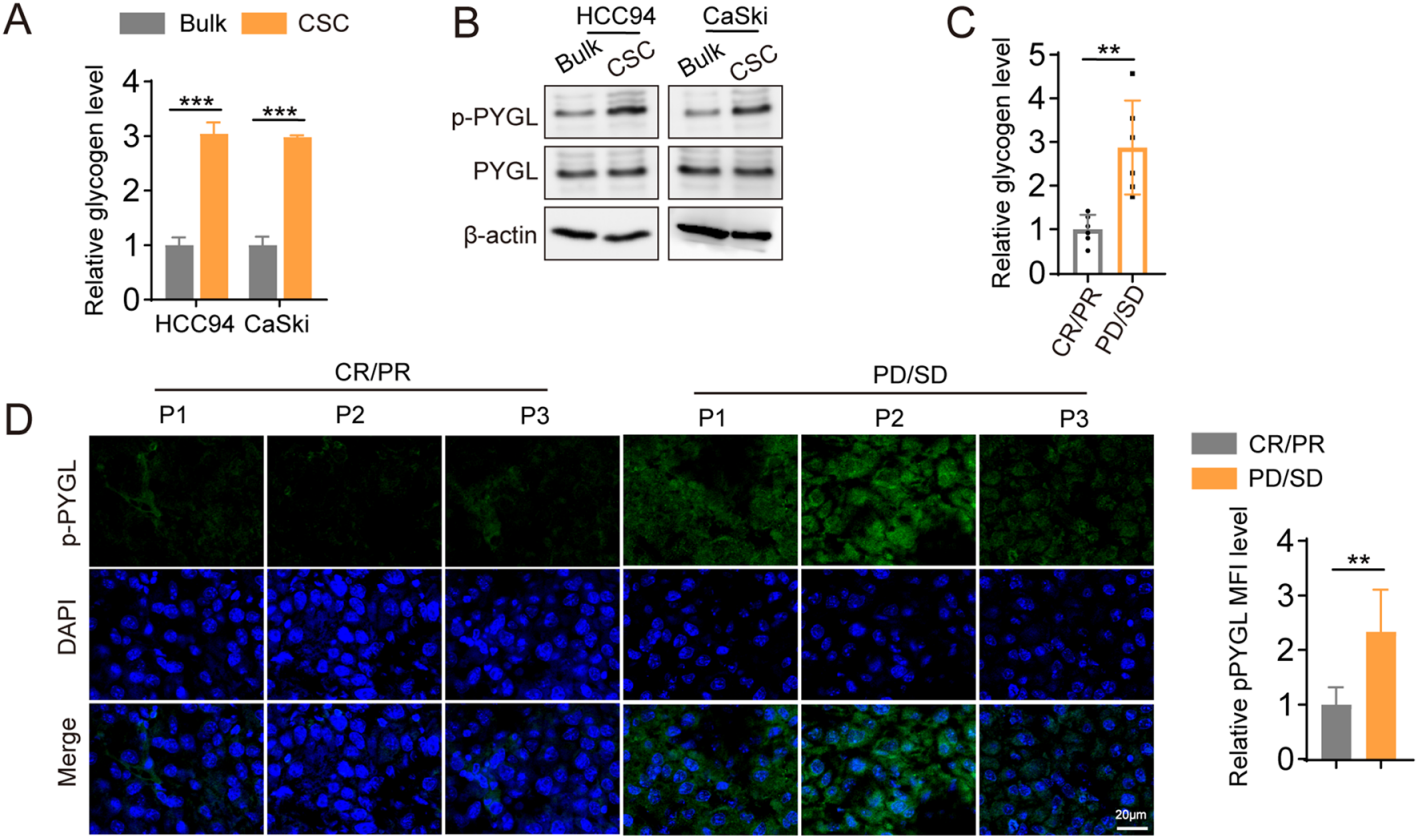
(**A**–**B**) HCC94 and CaSki human cervical cancer cells then sorted into two fractions: CD133^+^ (CSC), CD133^−^ (bulk cell). Glycogen levels was measured (**A**). The expression of p-PYGL and PYGL in CSC or bulk cell was measured (**B**). (**C**) The level of glycogen was measured in patients with chemotherapy-resistant or -sensitive cancers. (**D**) The level of p-PYGL was measured in patients with chemotherapy-resistant or -sensitive cancers by IF. In (**C**–**D**), n = 6 patients. The data were presented as means ± SEMs of three independent experiments. ***P < 0.001. Two-tailed student’s *t*-test (**A**, **C**, **D**).

Previous reports have indicated that glucose-6-phosphate (G6P), generated from glycogen, can predominantly flow into the pentose phosphate pathway (PPP)^8,15–17^. We discovered that glycogen levels and highly active phosphorylated glycogen phosphorylase (pPYGL) were upregulated in both cancer stem cells (CSCs) (Fig. 2A, B) and in patients with clinical chemotherapy resistance (Fig. 2C, D). The phosphorylation of PYGL plays a crucial role in regulating its activity and glycogen metabolism. When PYGL is phosphorylated, its enzymatic activity increases, leading to enhanced glycogen breakdown. This suggests the presence of elevated glycogen levels in CSCs, and the high level of phosphorylated PYGL leads to glycogen breakdown, generating G6P, which enters the PPP.

**Figure 3:**
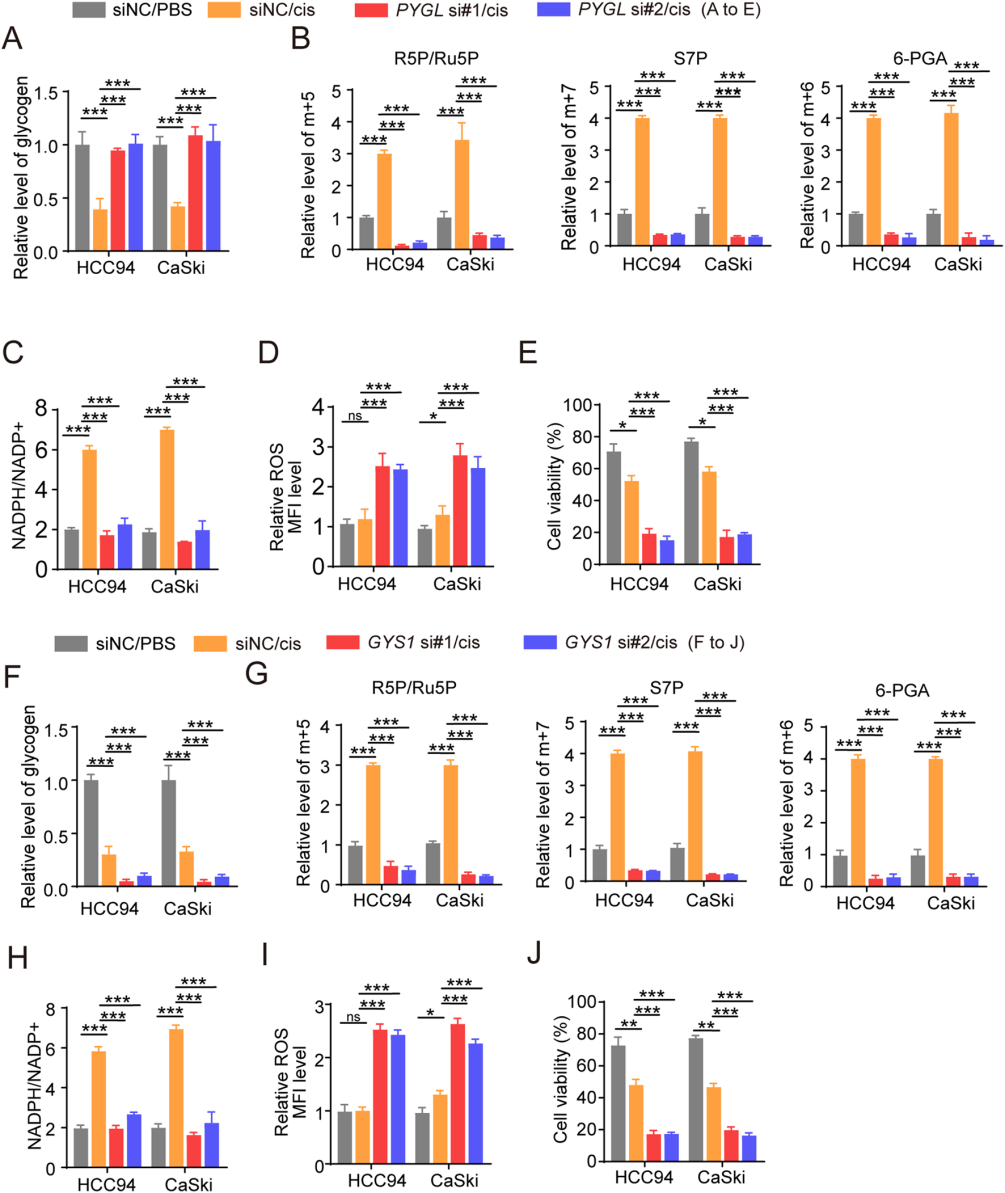
(**A**–**E**) HCC94 and CaSki human cancer stem cells, which was transfected with PYGL siRNA, were treated with PBS or cisplatin for 48 h. The level of glycogen (**A**), m+5 R5P, m+7 S7P, m+6 6-PGA (**B**), NADPH/NADP^+^ ratio (**B**), ROS (**C**) and cell viability (**D**) was measured. (**F**–**J**) HCC94 and CaSki human cancer stem cells, which was transfected with GYS1 siRNA, were treated with PBS or cisplatin for 48 h. The level of glycogen (**F**), m+5 R5P, m+7 S7P, m+6 6-PGA (**G**), NADPH/NADP^+^ ratio (**H**), ROS (**I**) and cell viability (**J**) was measured. The data were presented as means ± SEMs of three independent experiments. *P < 0.05, **P < 0.01, ***P < 0.001. One-way analysis of variance (ANOVA) followed by Bonferroni’s test (**A**–**H**).

During chemotherapy, knocking out PYGL in CSCs blocks glycogen breakdown, which disrupts the carbon flow towards 6-phosphogluconic acid, R5P/Ru5P, and S7P and NADPH production (Fig. 3A–C and Fig. S1B), resulting in a sharp increase in ROS levels (Fig. 3D) and disappearance of cellular resistance to cisplatin (Fig. 3E). Additionally, consistent results were obtained when glycogen synthesis was inhibited by GYS1 siRNA (Fig. 3F–J and Fig. S1C). In conclusion, these findings demonstrated that glycogen breakdown drives PPP in CSCs in response to drug treatment.

### PCK1 enhances the PYGL phosphorylation by competing with PP1c in cervical cancer

**Figure 4:**
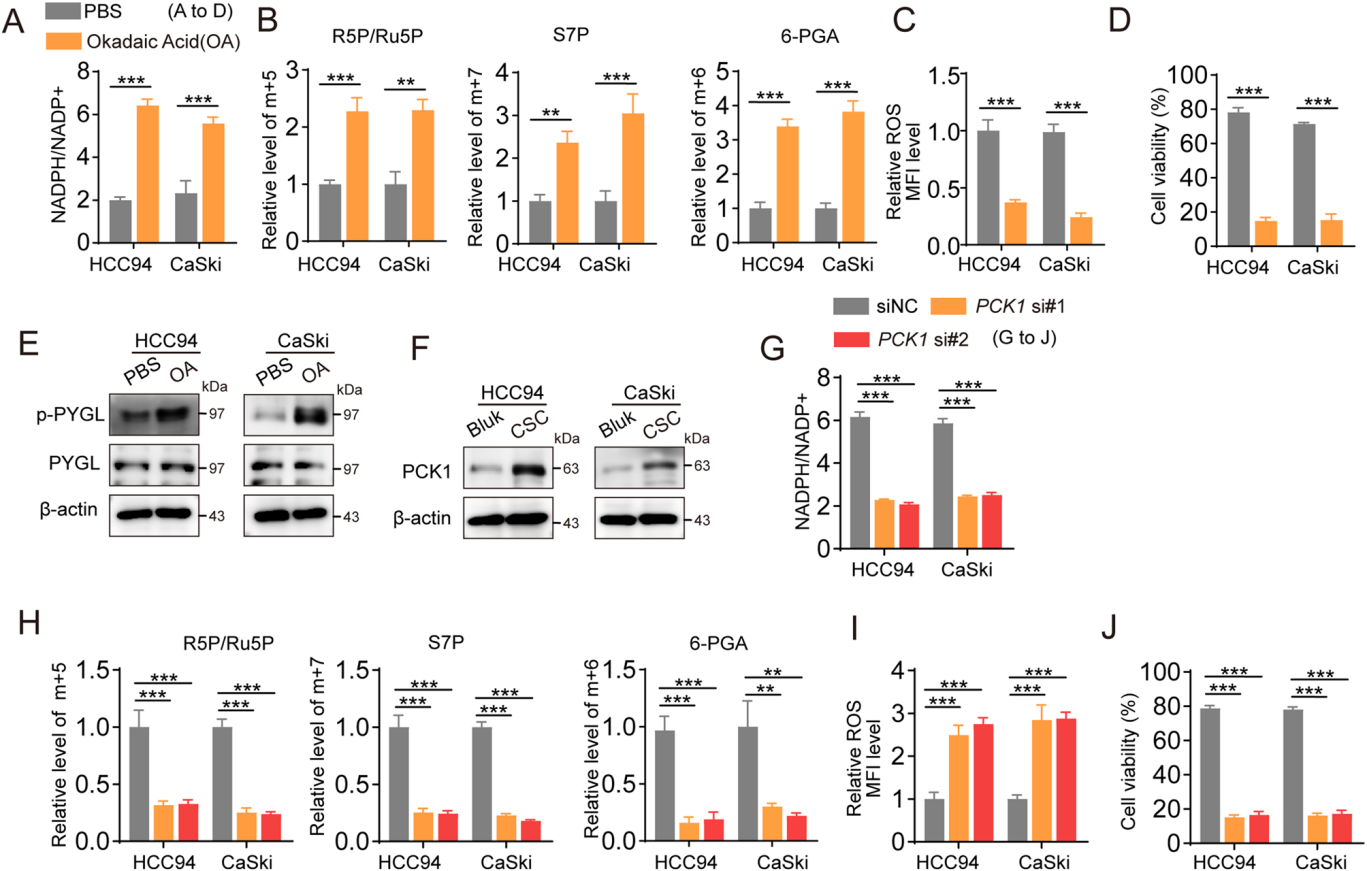
(**A**–**D**) HCC94 and CaSki human cancer stem cells, which was pretreated with okadaic acid (OA, 1 0 nM), were treated with cisplatin for 48 h. The level of NADPH/NADP^+^ (**A**), m+5 R5P, m+7 S7P, m+6 6-PGA (**B**), ROS (**C**) and cell viability (**D**) was measured. (**E**) The level of p-PYGL and PYGL was measured in HCC94 and CaSki human cancer stem cells, which was pretreated with OA. (**F**) The level of PCK1 was measured in HCC94 and CaSki human cancer stem cells or bulk cell. (**G**–**K**) HCC94 and CaSki human cancer stem cells, which was transfected with PCK1 siRNA, were treated with cisplatin for 48 h. The level of NADPH/NADP^+^ ratio (**G**), m + 5 R5P, m + 7 S7P, m + 6 6-PGA (**H**), ROS (**I**) and Cell viability (**J**) were measured. The data were presented as means ± SEMs of three independent experiments. **P < 0.01, ***P < 0.001. One-way analysis of variance (ANOVA) followed by Bonferroni’s test (**A**–**D**, **G**–**J**).

Next, we investigated the specific molecular mechanisms by which CSCs control glycogen breakdown, leading to chemoresistance. Phosphorylation of PYGL, a key enzyme involved in glycogen metabolism, is regulated by various signaling pathways and enzymes^10^. PYGL phosphorylation is controlled by kinases, such as PKA, PKC, and AMPK, as well as phosphatases, including PP1 and PP2A^18^. To explore the role of PYGL phosphorylation in chemoresistance, we pretreated bulk tumor cells with Okadaic acid, a specific inhibitor of PP1 and PP2A phosphatases. We observed that these bulk cells developed a drug-resistant phenotype similar to that of CSCs (Fig. 4A–D), which was accompanied by a significant increase in PYGL phosphorylation (Fig. 4E). This finding suggests that phosphorylated PYGL is a critical factor in chemoresistance. PCK1, a key enzyme involved in glucose metabolism, is highly expressed in melanoma cancer stem cells^19^. Interestingly, we also observed elevated expression of PCK1 in cervical CSCs (Fig. 4F). When we knocked out PCK1 in CSCs and subjected them to drug treatment, we observed reduced NADPH production, increased ROS levels, and loss of the drug-resistant phenotype (Fig. 4G–J). These findings suggest that PCK1 plays a crucial role in mediating chemoresistance in cervical CSCs through its effect on NADPH and ROS regulation.

**Figure 5:**
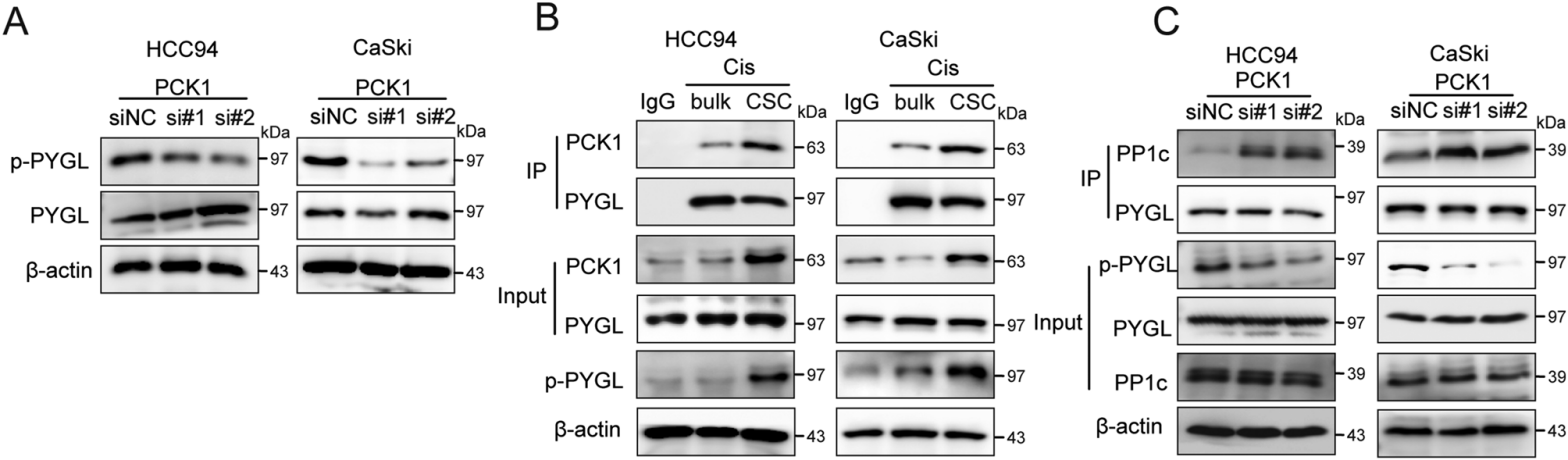
(**A**) HCC94 and CaSki human cancer stem cells, which was transfected with PCK1 siRNA, were treated with cisplatin for 48 h. The level of p-PYGL and PYGL was measured (**A**). (**B**) Immunoblot of immunoprecipitations of PYGL or PCK1 in lysates from cancer stem cells or bulk cells treated with cisplatin for 24 h. (**C**) Immunoblot of immunoprecipitations of PP1c or PYGL in lysates from cancer stem cells was transfected with PCK1 siRNA.

Moreover, we observed a significant reduction in PYGL phosphorylation upon PCK1 knockout (Fig. 5A and Fig. S2A). To further validate these findings, we conducted immunoprecipitation experiments and found that in CSCs, compared to bulk cells, the interaction between PCK1 and PYGL increased significantly (Fig. 5B), while the binding of PYGL to PP1c decreased. Additionally, in PCK1-silenced CSCs, the interaction between PP1c and p-PYGL increased, but the binding of PYGL to PP1c was reduced (Fig. 5C). Overall, these results suggest that PCK1 enhances PYGL phosphorylation by competing with PP1c.

### PCK1 regulated glycogenolysis promotes chemoresistance in vivo

**Figure 6:**
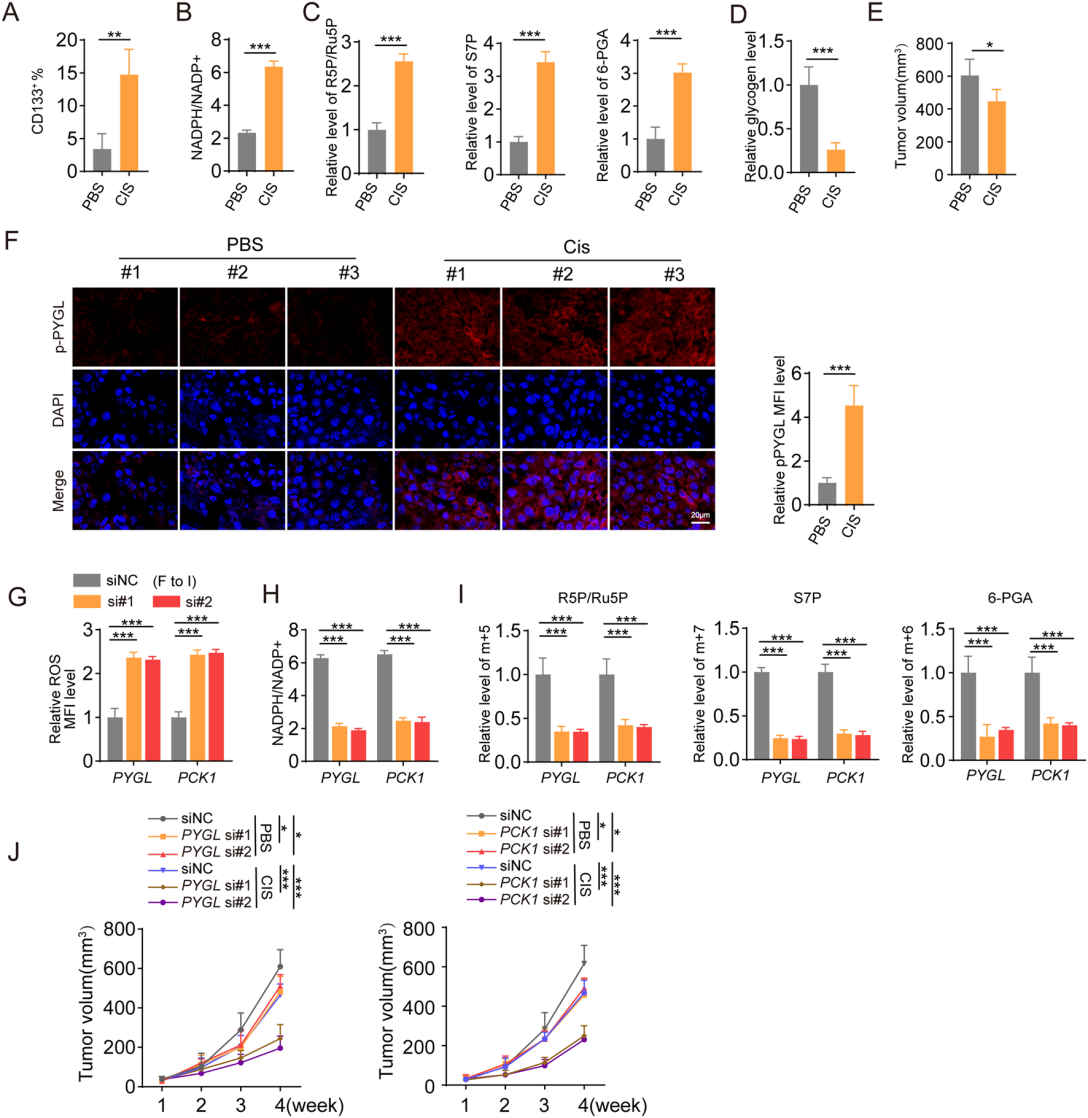
(**A**–**E**), HCC94 tumor-bearing mice were treated with cisplatin or PBS for 4 weeks. CD133 level (**A**), NADPH levels (**B**), the level of R5P, S7P, 6PGA (**C**), glycogen (**D**) and tumor volume (**E**) was measured. The expression of p-PYGL and PYGL was measured (**F**). (**G**–**J**) HCC94 tumor-bearing mice were treated with PBS or cisplatin and transfected with PYGL or PCK1 siRNA for 4 weeks. ROS level (**G**), NADPH levels (**H**), the level of R5P, S7P, 6PGA (**I**) and tumor volume (**J**) was measured. In (**A**–**J**), n = 6 mice. The data were presented as means ± SEMs of three independent experiments. *P < 0.05, **P < 0.01, ***P < 0.001. Two-tailed student’s *t*-test (**A**–**E**) or one-way analysis of variance (ANOVA) followed by Bonferroni’s test (**F**–**I**).

Next, we investigated whether these in vitro findings could be confirmed in vivo. For this purpose, we implanted cervical cancer cells into NSG mice and treated them with either cisplatin or PBS. We observed a significant increase in the proportion of CD133-positive stem-like cells and higher levels of NADPH and PPP intermediate metabolites such as 6-phosphogluconic acid, R5P/Ru5P, and S7P in the chemotherapy group than in the PBS group (Fig. 6A–C). Furthermore, we found decreased levels of glycogen and phosphorylated PYGL in the tumor tissue following chemotherapy, which was accompanied by a reduction in tumor volume. (Fig. 6D, E). Depletion of PYGL and PCK1 in the tumor tissue resulted in enhanced sensitivity to cisplatin chemotherapy, characterized by increased levels of ROS, reduced production of NADPH or PPP intermediate metabolites, and smaller tumor sizes (Fig. 6F–J). In conclusion, these findings demonstrate PCK1 regulated glycogenolysis promotes chemoresistance in a mouse model.

## Discussion

Chemotherapy resistance can arise through multiple mechanisms including increased drug efflux, enhanced drug metabolism, alterations in drug targets, abnormalities in cell death pathways, enhanced DNA repair, and altered cell cycle progression^20,21^. Tumor cells may upregulate efflux pumps, metabolizing enzymes, and DNA repair pathways, making them less susceptible to drug effects^22–24^. They may also exhibit changes in apoptotic signaling or cell cycle progression, thereby reducing drug efficacy^25,26^. Understanding these mechanisms is crucial for developing strategies to overcome drug resistance and improve treatment outcomes. Approaches such as identifying new targets, combination therapies, and personalized medicine hold promise in combating chemotherapy resistance. In this study, we identified a molecular pathway mediated by ROS clearance that contributes to chemoresistance. Upon entering tumor cells, drug molecules induce a significant increase in ROS production, triggering the PCK1-glycogen phosphorylase-glycogenolysis-PPP pathway, leading to NADPH generation and subsequent ROS clearance, ultimately resulting in chemoresistance.

PYGL has emerged as a crucial player in tumor biology. Dysregulation of PYGL has been implicated in various cancers, including breast cancer, head and neck squamous cell carcinoma, and liver cancer^10,27^. Elevated PYGL expression promotes glycogen breakdown, providing a source of energy for tumor growth and survival^28^. We found that PYGL activation contributed to chemoresistance in stem-like cells and enhanced CSC survival. Targeting PYGL and its associated pathways may have therapeutic potential for cancer treatment. However, further investigations are needed to fully understand the mechanisms underlying the role of PYGL in tumor development and to explore its clinical implications as a target for intervention.

Our findings initially showed an increase in glycogen levels in CD133^+^ cancer cells, which could fuel PPP for NADPH production, which is crucial for counteracting the oxidative stress induced by cisplatin. However, subsequent animal studies have revealed a decrease in glycogen levels, which better aligns with the expected metabolic adaptations to chemotherapeutic stress. Additionally, we observed an increase in phosphorylated PYGL, an indicator of glycogen breakdown, in vivo. These findings underscore the dynamic and complex nature of cellular responses to chemotherapeutic agents, and highlight the intricate metabolic reprogramming that cancer cells undergo to develop resistance mechanisms. This dual observation of glycogen dynamics emphasizes the variability of cellular stress responses and contributes to a deeper understanding of the biochemical pathways involved in cancer resistance.

PCK1 is an enzyme involved in glucose metabolism, and has been implicated in cancer development^29^. Its overexpression has been observed in various cancers, including melanoma and breast cancer, and is associated with tumor progression and poor prognosis^16,30^. PCK1 may contribute to tumor growth by providing energy substrates and intermediates for biosynthesis^31^. Additionally, PCK1 is involved in cancer stem cell properties and therapy resistance^32^. Targeting PCK1 shows promise as a therapeutic strategy; however, further research is needed to understand its precise role and therapeutic potential in different cancer types.

**Figure 7:**
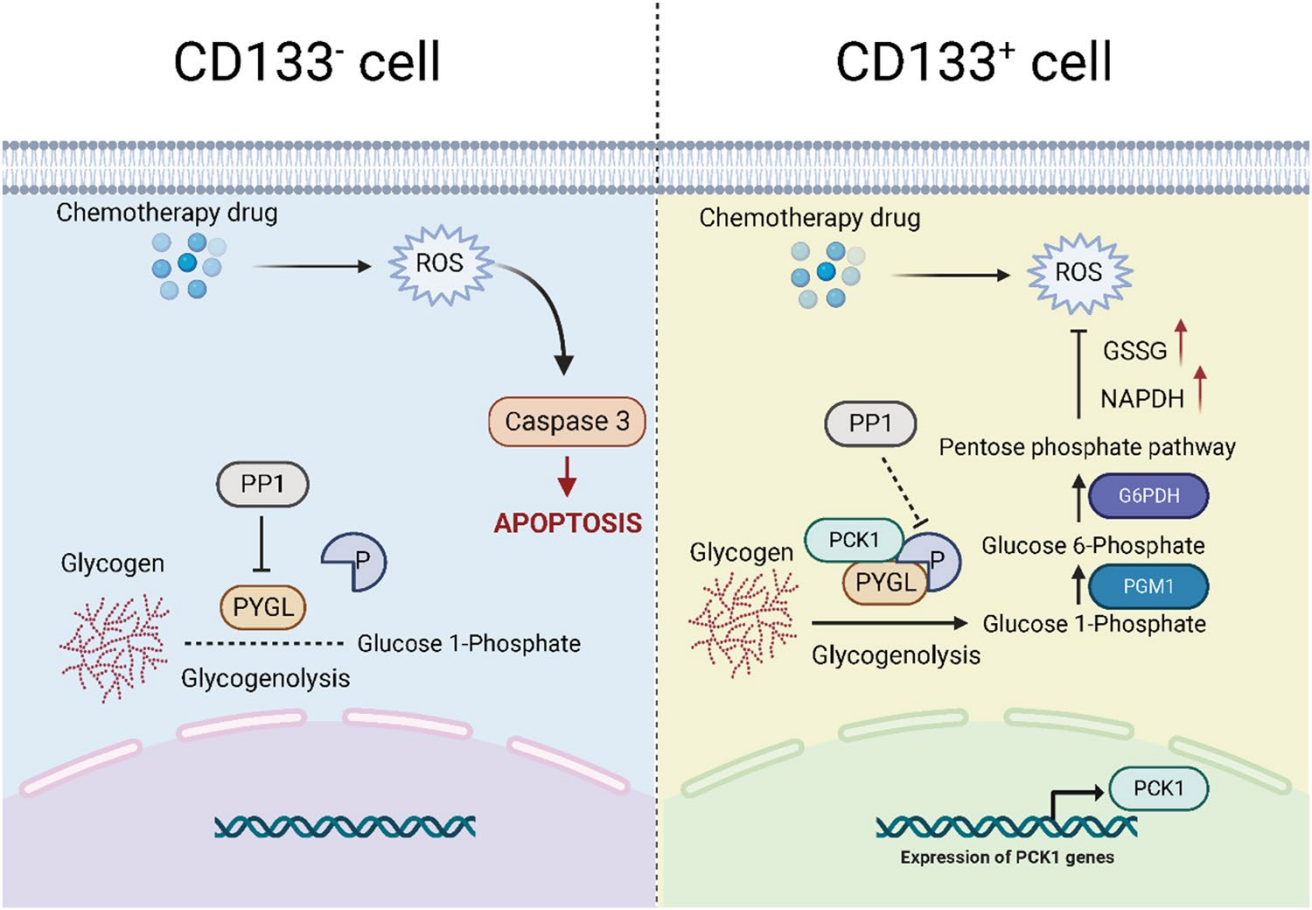
Proposed mechanism of PCK1-mediated glycogenolysis facilitates ROS clearance and chemotherapy resistance in cervical cancer stem cells. In drug-resistant tumor stem cells, the PCK1 enzyme promotes phosphorylation of PYGL, facilitating glycogen breakdown and directing the glucose flow towards the pentose phosphate pathway (PPP). This pathway generates NADPH, contributing to ROS clearance and cell survival.

Understanding the mechanisms underlying chemotherapy resistance involving ROS clearance, PYGL, and PCK1 opens new avenues for targeted interventions (Fig. 7). Future studies should explore combination therapies and personalized medicine approaches to overcome resistance and improve treatment outcomes in cancer patients.

### Supplementary Information

Supplementary Information.

## Data Availability

Upon reasonable request, the corresponding author will provide access to the datasets generated or analyzed during the current study.
