# Supplementary material for: PCK1-mediated glycogenolysis facilitates ROS clearance and chemotherapy resistance in cervical cancer stem cells
Source: Sci Rep. 2024 Jun 13;14:13670. doi: 10.1038/s41598-024-64255-6 (PMC11176388; doi:10.1038/s41598-024-64255-6)
Supplement: Supplementary file 1 — Supplementary Information. [file 41598_2024_64255_MOESM1_ESM.docx]

**Table 1 Patient information**

| Patient nubmer | Sex | Age | Location | chemotherapy resistance | T stage | Overall stage |
| --- | --- | --- | --- | --- | --- | --- |
| 1 | Male | ≤60 yr | Lower third | CR/PR | T2 | II |
| 2 | Female | >60 yr | Upper and mid | CR/PR | T3 | III |
| 3 | Male | >60 yr | Upper and mid | CR/PR | T4 | II |
| 4 | Male | ≤60 yr | Upper and mid | CR/PR | T2 | II |
| 5 | Male | ≤60 yr | Lower third | CR/PR | T3 | III |
| 6 | Female | ≤60 yr | Upper and mid | CR/PR | T2 | II |
| 7 | Female | ≤60 yr | Upper and mid | PD/SD | T3 | III |
| 8 | Male | ≤60 yr | Upper and mid | PD/SD | T3 | III |
| 9 | Male | ≤60 yr | Upper and mid | PD/SD | T3 | III |
| 10 | Male | ≤60 yr | Lower third | PD/SD | T4 | IV |
| 11 | Male | ≤60 yr | Lower third | PD/SD | T3 | IV |
| 12 | Female | >60 yr | Lower third | PD/SD | T3 | III |

Figure S1


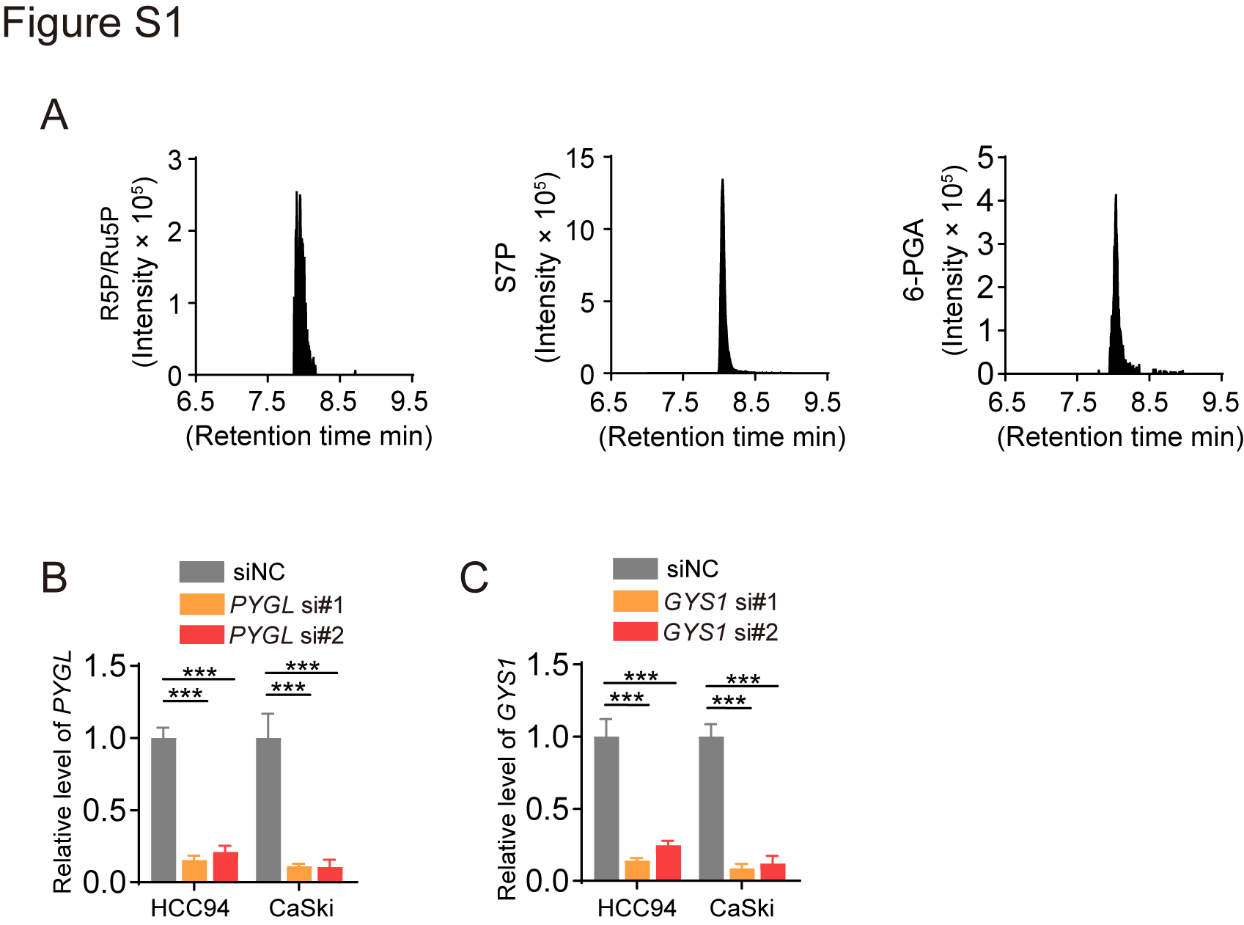


A, Mass spectrograms of R5P/Ru5P, S7P and 6-Phosphogluconic acid (6-PGA), levels by LC-MS in tumor cell.

.B, HCC94 and CaSki human cancer stem cells, which was transfected with PYGL siRNA, were performed for analyzing the efficiency of PYGL knockout. B, HCC94 and CaSki human cancer stem cells, which was transfected with GYS1 siRNA, were performed for analyzing the efficiency of GYS1 knockout. The data were presented as means ± SEMs of three independent experiments. ***P < 0.001. one-way analysis of variance (ANOVA) followed by Bonferroni’s test (A-B).

Figure S2


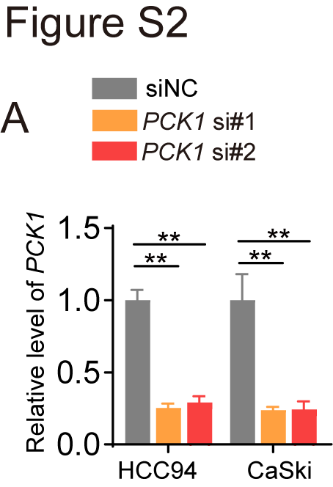


A, HCC94 and CaSki human cancer stem cells, which was transfected with PCK1 siRNA, were performed for analyzing the efficiency of PCK1 knockout. The data were presented as means ± SEMs of three independent experiments. **P < 0.01. one-way analysis of variance (ANOVA) followed by Bonferroni’s test (A).

Figure S3


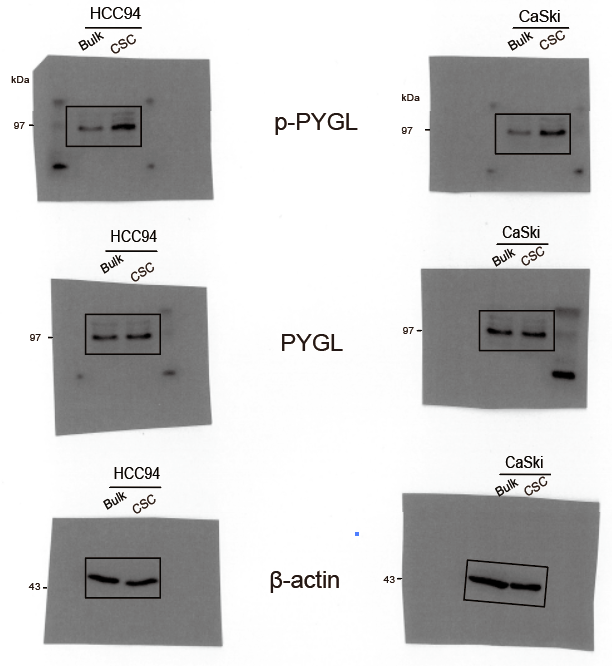


Uncropped images of immunoblots for figure 2B.


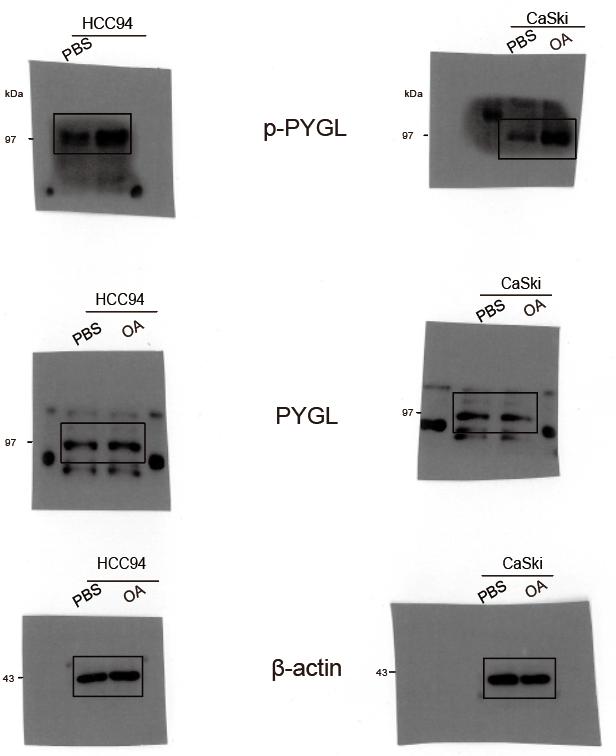
Figure S4

Uncropped images of immunoblots for figure 4E.


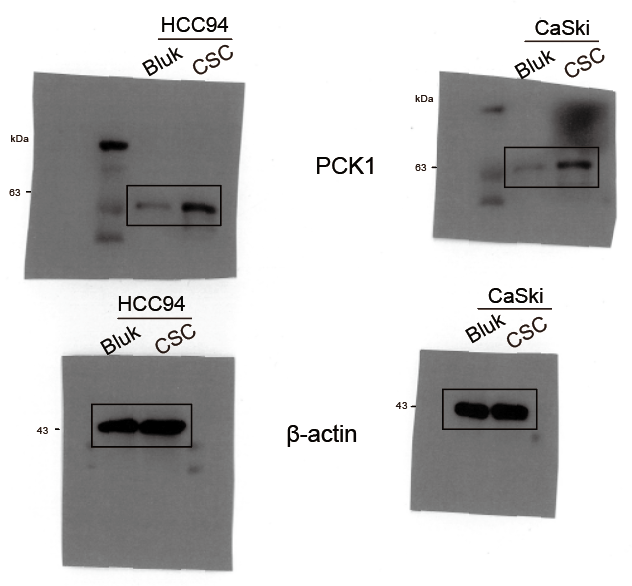
Figure S5

Uncropped images of immunoblots for figure 4F.


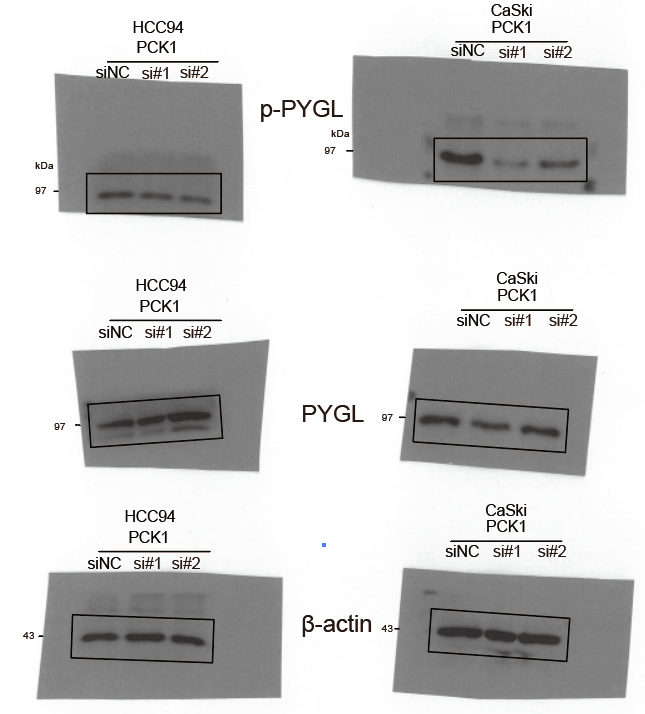
Figure S6

Uncropped images of immunoblots for figure 5A.


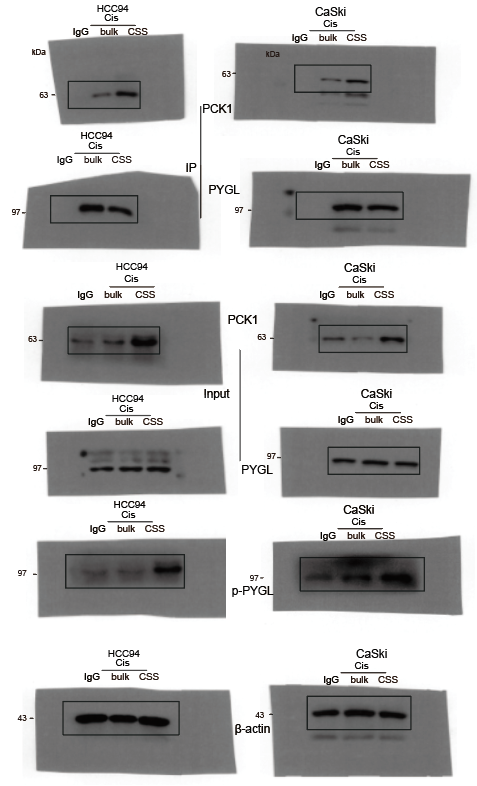
Figure S7

Uncropped images of immunoblots for figure 5B.


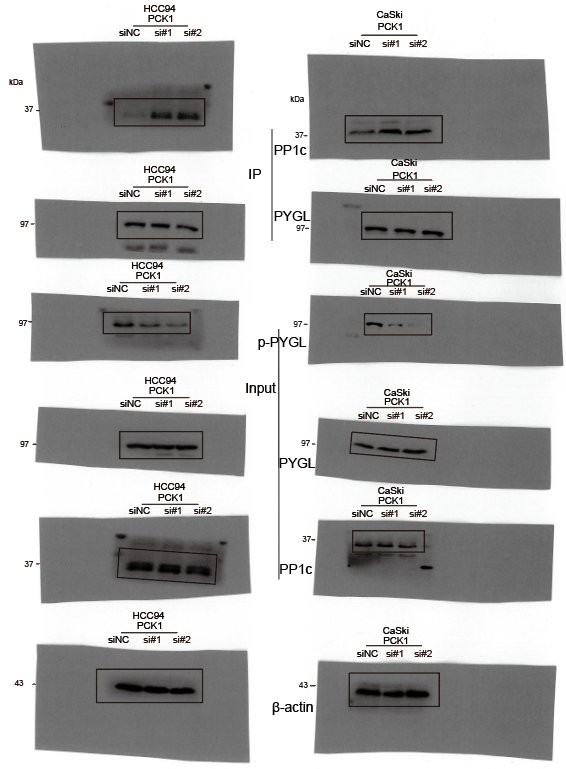
Figure S8

Uncropped images of immunoblots for figure 5C.
